# Describing Prehospital Deliveries in the State of Michigan

**DOI:** 10.7759/cureus.26723

**Published:** 2022-07-10

**Authors:** David Eisenbrey, Robert B Dunne, William Fales, Kristopher Torossian, Robert Swor

**Affiliations:** 1 Emergency Medicine, McLaren Oakland Hospital, Pontiac, USA; 2 Emergency Medicine, Wayne State University, Detroit, USA; 3 Emergency Medicine, Western Michigan University Homer Stryker MD School of Medicine, Kalamazoo, USA; 4 Emergency Medicine, Oakland University William Beaumont School of Medicine, Rochester Hills, USA; 5 Emergency Medicine, Oakland University William Beaumont School of Medicine, Troy, USA

**Keywords:** out-of-hospital delivery, paramedic, midwifery, emergency obstetrics, emergency medical services

## Abstract

Introduction

We observed clinically that prehospital deliveries locally appeared to have a high rate of complications and appeared associated with midwife deliveries. There is scant literature that addresses prehospital deliveries across a state. We set out to describe utilization, complications, and short-term outcomes of EMS-attended prehospital deliveries in Michigan in 2015, and to describe the relationship between prehospital delivery and socioeconomic status (SES).

Methods

We identified candidate cases for prehospital deliveries through the Michigan EMS Information System (MI-EMSIS). To assess the relationship of SES with the frequency of EMS delivery, we utilized the mean income of the patient residences' zip codes.

Results

We identified 223 EMS-attended deliveries from 1.6 million MI-EMSIS records. Most births were normal vaginal deliveries on the scene or en route to the hospital (92, 40.0%) or delivered prior to EMS arrival (58, 25.4%). Maternal or fetal complications were identified in 69 (32.0%) deliveries. We identified a few midwife-attended deliveries (31), but these had a high rate of complications (19, 61.3%). The frequency of prehospital delivery was inversely related to estimated patient income (Pearson=-0.85).

Conclusions

EMS deliveries were rare and most were normal vaginal deliveries, but almost a third had complications. Midwife and EMS-attended deliveries were rare, but when they occurred, had high rates of complications. Although an imperfect measure of patient SES, frequency of delivery was inversely related to patient income, and agencies that provide care in these communities should have focused training.

## Introduction

Out-of-hospital (OOH) emergency deliveries are widely perceived by emergency medical service (EMS) providers as either a joyful and unique experience or one of the most terrifying experiences in their career [1]. Observations at our institution have suggested that emergency deliveries were among the most difficult emergency cases transported to our facility, involving at least two patients (mother and most often one child) for which EMS providers were poorly prepared. There is an ongoing perception among emergency medical service providers that childbirth is one of the more stressful and challenging operational situations in which they find themselves [1]. OOH births are uncommon, with data from a large national cardiac arrest dataset identifying that OOH births are far less likely than other relatively rare OOH events such as cardiac arrest [2].

Previous literature has also identified that prehospital deliveries are more frequent in those communities with lower socioeconomic status (SES) [3]. Several retrospective studies had been conducted in multiple locations worldwide with the aim of exploring the morbidity and mortality of prehospital provider-attended birth [4-7]. Complication rates have varied widely among various reports with a recent meta-analysis indicating 20-60% morbidity [6-10]. EMS providers across the United States vary in their training and experience, ranging from physicians to non-licensed providers [11]. There is a paucity of literature evaluating EMS providers’ experience with OOH delivery in the United States [1,6,8].

An OOH delivery is not an uncommon phenomenon in the United States, with many ethnic communities, cultures, and individuals opting for home deliveries by midwives. The purpose of a midwife is to be present at the delivery of a child at home and to provide a delivery in a non-medicalized environment, reduce costs, and provide a more naturalistic setting for delivery. When this is done and coupled with appropriate prenatal care, it has been shown to be safe and effective [7-8,12-13]. Conversely, if not coupled with prenatal care, outcomes for mother and child have been shown to be worse than delivering in a hospital [14-15]. In 15 states, midwives (not nurse-midwives who operate under their nursing license) are not required to be licensed or certified [16]. This increases the variation in both prenatal and perinatal care, with the potential for poor prenatal screening and failure to identify potential complications such as abnormal presentations, congenital anomalies, or even multiple gestations.

When birth complications arise, and the mother or child requires emergency care, EMS providers are summoned to provide emergency care and transport. In a literature search of the current global studies focused on EMS providers’ experience with childbirth, we found scant literature that addresses this difficult circumstance [5,9] and no studies at all that discuss the interaction between EMS and midwives. For these reasons, our objectives in this study were to describe the utilization, complications, and short-term outcomes of EMS-attended pre-hospital deliveries in Michigan in 2015, the frequency and nature of the complications of midwife-attended OOH deliveries, and the relationship between prehospital delivery and SES.

## Materials and methods

**Figure 1:**
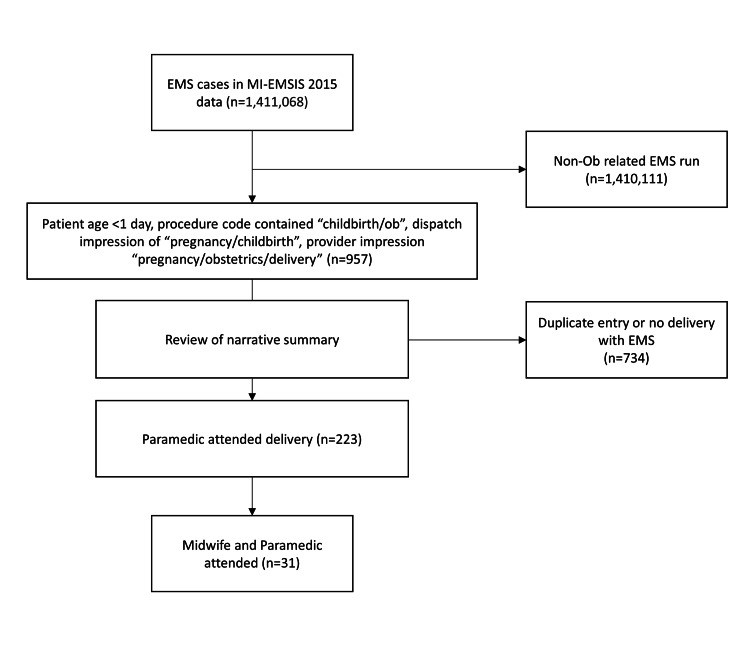
Case Selection Tree

We performed a cross-sectional study by accessing the Michigan EMS Information System (MI-EMSIS) v. 2.0 during 2015 for prehospital deliveries. The data were selected for 2015 due to completeness and due to the law changing for midwife licensing and certification in 2017 (Figure 1). Although the rules did not get finalized and put into effect until 2019, we wished to avoid any confounding from the law changing the pattern of care. Institutional review board approval was obtained from the Michigan Department of Health and Human Services (MDHHS IRB Log #: 201312-05-EA). The MI-EMSIS database is a partner of the National EMS Information System (NEMSIS). NEMSIS is a national database that is used to store data for US states and territories and provide surveillance regarding EMS care [17]. During this study period, MI-EMSIS received EMS records from agencies that provided care to 90% of the population of the State of Michigan. Because there is no single diagnostic code for that reliably identified OOH delivery, we utilized multiple search strategies to identify prehospital births. We identified candidate cases using a combination of narrative, demographic, and procedural search strategies.

This included sorting for patient age (< 1 day), procedure code of “childbirth/ob”, dispatch impression of “pregnancy/childbirth”, or provider primary or secondary impression of “pregnancy/obstetrics/delivery”. Cases were included in the analysis only if birth was identified as being prior to hospital arrival in the runsheet narrative. All other cases that did not meet the above inclusion criteria were excluded. We abstracted demographics, birth circumstances, complications, and short-term birth outcomes from run sheet data. We also abstracted from the EMS narrative whether or not there was a midwife present on the scene. We conducted duplicate reviews of 20% of the cases to assure interrater reliability of determination of case inclusion and complications. As we were uncertain which categories of complications would be the most pertinent, categories were constructed post hoc. In accordance with the American College of Obstetricians and Gynecologist Clinical Guidelines and the U.S. National Center for Health Statistics, we defined a non-viable fetus as one delivered prior to 20 weeks gestation based on documentation in the narrative [18]. Fetal demise was defined as historical evidence of pre-delivery absence of fetal activity or a delay between delivery and EMS arrival preventing resuscitative care. Extreme prematurity was defined as delivery of > 20 weeks gestation and < 37, but with a child that appeared viable [18]. We only assigned one category of complication per delivery. To assess the relationship of SES with the frequency of EMS delivery, we utilized the mean income of the patient's residence zip code. The University of Michigan Population Studies Center dataset was abstracted for mean and median national income and then further refined for mean and median income for Michigan’s zip codes [19]. Individual patient data were matched to the dataset. Income brackets were stratified by $10,000 increments starting with <$40,000 and continuing up to $100,000 and greater. Descriptive statistics were used to describe means and frequencies. We used Pearson’s correlation coefficient to assess relationships between the median of each income bracket, birth frequency, and rates of complications (Microsoft Excel, 2013, Microsoft Corporation, Redmond, WA). A kappa statistic was calculated to measure agreement by reviewers for the identification of emergency delivery.

## Results

In 2015, Michigan Vital Statistics reported 1,577 OOH (excluding birthing center) births [20]. MI-EMSIS received approximately 1.6 million EMS records during the calendar year 2015, 1.4 million of which were ambulance responses. We reviewed 957 cases that met one of the above-referenced search terms and ultimately identified 223 unique EMS-attended deliveries. Agreement regarding the classification of these cases was excellent (Kappa = 0.84). The mothers’ median age was 28 (range 15-41). The majority with race identified were Caucasian (64.4%) or African-American/Black (30.0%).

Most deliveries were uncomplicated vaginal deliveries on scene or en route to hospital (91, 40.8%) or delivered prior to EMS arrival (55, 24.7%). Maternal or fetal complications were identified in 69 (32.0%) of deliveries. Fetal complications included non-viable fetus (delivery before 20 weeks gestation (13, 5.8%), critically ill neonates (neonatal demise) (11, 4.9%; 1, 0.4%), CPR (1, 0.4%), non-transient apnea (5, 2.2%), and extreme prematurity (6, 2.7%). Birth-related complications included abnormal presentation or postpartum hemorrhage (Table 1).

**Table 1:** Complications in All Cases of Prehospital Delivery

Complications - All Cases	N	%
Non-Viable Fetus	13	5.8%
Fetal Demise	10	4.9%
Post-Partum Hemorrhage Including Vaginal Tears	10	4.9%
Abnormal Presentation Congenital Anomaly	7	3.1%
Non-Transient Apnea Including CPR	6	2.2%
Extreme Prematurity	6	2.7%
Circumnuchal Cord	5	2.2%
Multiple Gestations	3	1.3%
Other	9	4.0%

We identified a small number of midwife-associated cases (N=31), but these had a relatively high proportion of complications (N=19, 61.3%) (Table 2).

**Table 2:** Complications of Midwife Prehospital Delivery

Complications of Midwife Cases	Midwife Complications (19)	% of Midwife Cases (32)	% of Total Cases
Fetal Demise	2	6.3%	0.90%
Post-Partum Hemorrhage Including Vaginal Tears	8	25.0%	3.60%
Abnormal Presentation of Congenital Anomaly	2	6.3%	0.90%
Non-Transient Apnea Including CPR	4	12.5%	1.80%
Others	3	9.4%	1.30%

**Figure 2:**
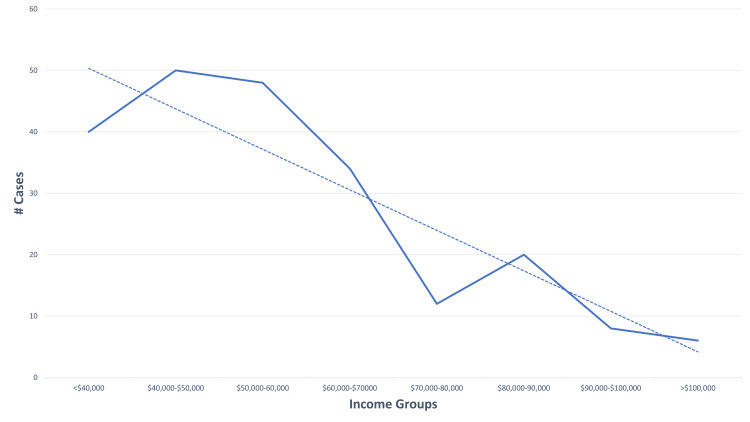
SES Status and Frequency of EMS-Attended Deliveries SES: socioeconomic status; EMS: emergency medical service

The frequency of prehospital delivery was inversely related to estimated patient income (Pearson=-0.85) (Figure 2). Complication rates were also inversely associated with estimated patient income (Pearson =-0.84).

## Discussion

We identified that EMS-attended deliveries were rare and that most were uncomplicated vaginal deliveries either upon or before EMS arrival on the scene. However, 31% had complications. These complication rates compare to previously reported literature where 34% of deliveries had complications [5,9]. While some complications were not amenable to treatment (e.g. non-viable fetuses), others (extreme prematurity, non-transient apnea, abnormal presentations) represented severe clinical challenges for EMS personnel. We identified that midwife-associated EMS deliveries were infrequent in our dataset, comprising only 32 (14.0%) of identified deliveries, but this small number corresponded to a higher proportion of complications at delivery. Finally, although an imperfect measure of patient SES, frequency of delivery was inversely related to mean patient income as determined by zip code.

These data identify those deliveries that occur OOH and those that are attended by EMS providers present a difficult care environment. Obstetrical emergencies are very rare (N=223) when compared to other OOH emergencies. For sake of comparison, during 2015, the Michigan CARES registry reported 4,767 adult cardiac arrests, documenting that an EMS provider is 20 times more likely to have experience with cardiac arrest than a prehospital delivery. While the majority of the deliveries in this dataset required only supportive care, as reported in the narrative summaries by the EMS providers, those with complications required the management of a mother, a neonate, or both. Pre-term deliveries are especially daunting, in that ambulances may not be equipped to provide basics of care, such as appropriate-sized masks to perform bag valve mask ventilation or endotracheal tubes. The paucity of experience EMS providers may have with deliveries is not addressed by focused didactic and hands-on education. As an example, we compare EMS education for cardiac vs obstetrical emergencies in Michigan, whose standards are based on the National Standard Curriculum [21-23], and identify very limited education on this topic (Table 3). Although our data regarding the incidence of events by income is not robust, it does document an increased rate of OOH birth in communities with lower socioeconomic status, supporting a particular need for prenatal education.

**Table 3:** Training Requirements in the State of Michigan 2020 ED: emergency department; ICU: intensive care unit; TCU: transitional care unit; OB: obstetrics

Training Requirements in the State of Michigan 2020 (Michigan Department of Health and Human Services – EMS Education Coordination Office)	Cardiac Emergencies	Obstetrical Emergencies
Basic (Must have Health Care Provider CPR)	16 hours	4 hours (didactic and psychomotor)
Paramedics (Must Have Health Care Provider CPR)	64 hours	10 hours
Paramedic Clinical Hours	250 hours ED, ICU, TCU, Surgery, and OB; OB requirement is only 10 patient exams	

Our initial observations suggest that midwife-attended births might well be a particularly complex circumstance for EMS providers and their patients. We theorize that, unlike most circumstances where EMS is called to a scene, EMS providers are comparatively poorly trained and equipped to handle an obstetrical complication (Table 3) and further arrive at an emergency that is attended by a health care provider who presumptively has a wealth of obstetrical experience. The transport of a stalled delivery or child in distress alone is among the most difficult circumstances that any health care provider might face [1], much less one with minimal obstetrical experience. The potential for disaster is great, particularly when prenatal screening may not have been performed. Licensure, certification, and practice of midwifery in the United States are not uniform, with many states (such as Michigan during this study period) not requiring a license to practice in the community [16]. Our data only identified two midwife-managed deliveries that were complicated by congenital anomalies that were not addressed prior to a home delivery and another three with neonatal demise. We suggest that the evaluation of prehospital delivery data might be an effective public health tool for monitoring safe deliveries in the community. A review of this data may be a useful tool to identify gaps in the care of OOH deliveries.

Limitations

This study has limitations driven primarily by its data source. Data in narrative summaries written by EMS providers and collected in the EMSIS database was not structured, leading to a variation in the quality of data regarding resuscitative efforts or prenatal care.

The EMSIS dataset was limited in that there was no single variable that reliably identified both a mother who just delivered and the infant. Some records combined documentation of both patients while others cases were documented with two records. Methods of uniform documenting of such events should be standardized in the future, such that both patients always have their own records. We felt confident in our ability to find and re-link the records of mothers and neonates, but this was accomplished by reading individual record narratives. This method would not be able to be used in larger national datasets. While our research into the interactions between EMS providers and midwives is novel, this too was limited, depending on provider narratives to identify the presence of a midwife. Our data set was not able to identify imminent deliveries that may have occurred shortly after hospital arrival and yet would be significantly influenced by the EMS-provided stabilization and transport. Strategies to link EMS and hospital records, such as unique identifiers that allow deterministic linkage would be important to further expand this area of inquiry.

As has been reported in numerous publications, zip code, as a measure of SES, is imperfect and can cover significantly heterogeneous areas, losing the granularity that is required to make broad statements about public health, epidemiology, and EMS data.

## Conclusions

EMS deliveries were rare and most were normal vaginal deliveries, but almost a third had complications. Midwife-attended deliveries were rare; however, when they occurred they had high rates of complications. This data suggest that EMS deliveries are rare and generally uncomplicated but may present EMS providers with very difficult circumstances for which they have little experience. EMS data may be a useful tool to monitor and identify gaps in care for those patients that have an OOH delivery.
